# Treatment of Rigidity of the Os Uteri

**Published:** 1849-12

**Authors:** 


*Treatment of Rigidity of the Os Uteri.* By Dr. Scanzoni.—Dr. Scanzoni, who has carefully examined the conditions of the os and cervix, in the latter months of pregnancy, believes that the constriction, which sometimes declares itself in the first stage of labor, is due to rigidity of the upper orifice of the uterine neck, and not the lower, which is generally sufficiently dilatable. Instead of the treatment usually recommended, viz: bleeding, antimony, belladonna, frictions, &c., he advises a continuous douche of warm water upon the os and cervix, directed by means of an appropriate instrument.—*Union Medicale.*

[On the subject of the *artificial dilatation of the os uteri*, Dr. Lawrence of Montrose, observes:]

I would advert to two points of practice, of which my experience is decidedly in favor, although I cannot offer any statistics regarding them. The first is gentle dilatation of the os uteri with the finger, when the pains are at all inefficient. That uterine action is often rendered much more brisk and effective in this manner, I entertain not the smallest doubt; while, on the other hand, I have never experienced anything unfavorable to result from it.—Query—Does the oxytoxic effect depend upon a reflex action being excited by the pressure of the finger, or upon the os uteri being placed in a more favorable position for being acted on by the longitudinal fibres of the body of the organ, when the os is pushed a little forward—just as the eye, when it has been deeply inverted by the internal rectus cannot be advantageously acted on by the external rectus, until it is brought forward by the inferior oblique?

The second point of practice referred to, is the method adopted to secure timely expulsion of the placenta. Besides directing pressure to be made over the uterus after the birth of the child, I invariably lay hold of the cord, and, without pulling, *retain it in a state of tension.* This seems to have the effect of exciting the uterus to speedy contraction ; and to this practice I attribute the fact of the placenta being expelled in all my cases within ten or fifteen minutes, in the majority within five minutes after the birth of the child. While it cannot explain the fact of my fortunately having met with no cases of retained placenta from adhesion, I think I am warranted to ascribe to this method of procedure the fact of my having met with no cases of placental retention from inertia, or irregular contraction of the uterus.

I may also state what may seem almost too petty to mention, that I never use tape for tying the funis before the separation of the child, but always strong sewing thread. I was led to this from having got some serious frights at the outset of practice by the slipping of the tape, and the alarming, and to the child almost fatal hemorrhage which ensued. I have known the same casualty to occur in the practice of others, from the same cause. —*Monthly Journal.*

